# Parental smoking and child poverty in the UK: an analysis of national survey data

**DOI:** 10.1186/s12889-015-1797-z

**Published:** 2015-05-29

**Authors:** Charmaine Belvin, John Britton, John Holmes, Tessa Langley

**Affiliations:** Division of Epidemiology and Public Health, University of Nottingham, Nottingham, UK; UK Centre for Tobacco and Alcohol Studies, Nottingham, UK; School of Health and Related Research, University of Sheffield, Sheffield, UK

**Keywords:** Smoking prevalence, Child poverty

## Abstract

**Background:**

In 2011/12 approximately 2.3 million children, 17% of children in the UK, were estimated to be in relative poverty. Cigarette smoking is expensive and places an additional burden on household budgets, and is strongly associated with socioeconomic deprivation. The aim of this study was to provide an illustrative first estimate of the extent to which parental smoking exacerbates child poverty in the UK.

**Methods:**

Findings from the 2012 Households Below Average Income report and the 2012 Opinions and Lifestyle Survey were combined to estimate the number of children living in poor households containing smokers; the expenditure of typical smokers in these households on tobacco; and the numbers of children drawn into poverty if expenditure on smoking is subtracted from household income.

**Results:**

1.1 million children - almost half of all children in poverty - were estimated to be living in poverty with at least one parent who smokes; and a further 400,000 would be classed as being in poverty if parental tobacco expenditure were subtracted from household income.

**Conclusions:**

Smoking exacerbates poverty for a large proportion of children in the UK. Tobacco control interventions which effectively enable low income smokers to quit can play an important role in reducing the financial burden of child poverty.

**Electronic supplementary material:**

The online version of this article (doi:10.1186/s12889-015-1797-z) contains supplementary material, which is available to authorized users.

## Background

In 2011/12 2.3 million children in the UK, or 17% of all children, were living in relative poverty defined as less than 60% of median equivalised household income [1]. These children are more likely to live in inadequate housing and in more deprived communities, be exposed to high levels of air pollution, have a poor diet, develop depression and other long term health problems, and to be absent from school [2-4]. Growing up in poverty is thus a blight on child health and development. In 1999 the UK government announced a target of halving the number of children living in poverty by 2010, and abolition of child poverty by 2020. However, the 2010 target of 1.7 million was missed by 600,000 [5], and it is now unlikely that the 2020 target will be met. It is therefore important to identify avoidable factors that contribute to and exacerbate child poverty.

Tobacco smoking is powerfully addictive and strongly associated with socioeconomic deprivation [6], and is also a major cause of ill health. Passive exposure of children to tobacco smoke increases the risk of sudden infant death, respiratory infections, asthma and middle ear disease, and children growing up among smokers are twice as likely to become addicted to smoking themselves [7,8]. Smoking is thus a significant cause of poor health of children living in poverty [9]. However, it is also a direct contributor to financial deprivation. In January 2015 the weighted average price of 20 cigarettes in the UK was £7 [10], and although smokers can reduce the cost of smoking by opting for budget brands or switching to handrolling tobacco, the cost of regular smoking represents a significant burden on the budgets of families living on low incomes.

Existing studies have explored the financial impact of smoking in both high and low income countries. A study from New Zealand found that if second-lowest income decile households containing a smoker were to become smoker-free, on average, 14% of non-housing budgets in those households could be reallocated [11]. A study in the USA found that smokers spend less on housing than non-smokers [12]. Research from India has indicated that tobacco expenditure crowds out expenditure on food, education and entertainment, and that when both direct tobacco expenditure and out-of-pocket payments on tobacco-attributable medical care are taken into account, tobacco consumption impoverishes roughly 15 million people in India [13,14]. Evidence from Bangladesh has suggested that poor smokers could add over 500 calories to the diet of one or two children with their daily tobacco expenditure, and that tobacco prices are positively associated with child health outcomes [15,16]. To our knowledge, the impact of parental smoking on child poverty has not previously been estimated in the UK. This paper therefore aims to provide an illustrative first estimate of the number of children in poverty in the UK who have smoking parents, the possible cost of smoking in this context, and the number of children living above the poverty line, but who would fall below the poverty line if household resources were assessed after accounting for expenditures on tobacco.

## Methods

Our analyses combined findings from several national surveys, taking the most recent available at the time of the study, to estimate the number of children living in relative poverty by household structure; apply smoking prevalence data to estimate the number of children living in poor households containing smokers; and then estimate the expenditure of typical smokers in these households on tobacco. Finally we estimated the numbers of children drawn into poverty if expenditure on smoking is subtracted from household income. Where published survey sources did not provide data broken down into the required level of detail we used conservative assumptions to generate estimates. The study used publically available data and ethics approval and participant consent were therefore not required.

### Definition of poverty

Poverty was defined as living in a household with an equivalised net household income before housing costs (BHC) below 60% of the median equivalised net household income [1]. Equivalised income is the sum of income after deductions of income tax, employee and self-employed national insurance contributions and council tax for all household members, rescaled to allow for household composition, to reflect the fact that larger households need more income to maintain the same standard of living. The data were equivalised using the modified OECD equivalence scale, using an adult couple with no children as the reference point [17]. In 2011/12 the median equivalised household income per week was £427 BHC. Poverty was therefore defined as an equivalised income BHC of £256 or less [1].

### Numbers of children in poverty

We estimated numbers of children in poverty by household composition using data from the Department for Work and Pensions’ 2012 Households Below Average Income (HBAI) report [1]. This draws on data from approximately 20,000 households in the Family Resources Survey and provides estimates of the number of all children broken down by parental marital status, and the percentage of those children living in poverty. We combined these figures with data from the report on the proportion of poor households with one, two, or three or more children (calculated as 25%, 39% and 36% respectively) to estimate the number of children living in poverty by parental marital status and family size. A worked example of these calculations is provided in Additional file 1. In the HBAI report children are defined as those under 16, and those aged 16–19 who are dependent (living with parents and in full time education or in unwaged government training).

Since the HBAI report does not provide data on the proportions of single parents who are male and female, we used estimates of these proportions (9% and 91% respectively) from the Office for National Statistics’ (ONS) 2012 Families and Household survey to calculate the number of children living in poor households with a single mother and a single father [18].

### Smoking prevalence in poor households

To estimate the proportion of children in poverty with one or more parents who smoke, we first estimated parental smoking prevalence in these households using data from the 2012 Opinions and Lifestyle Survey [19]. Since the survey reports do not present smoking prevalence by poverty status, and no other relevant survey data were available, we made the conservative assumption that smoking prevalence in households in poverty would be the same as that in households in the routine and manual occupational socio-economic group. The prevalence of smoking among men and women in routine and manual occupations in Britain in 2012 was 33% and 32% respectively. In fact these figures are highly likely to underestimate smoking prevalence among the poor, as among unemployed people the prevalence is substantially higher (39% in 2012 [19]).

Since the 2012 Opinions and Lifestyle survey indicated that smoking rates vary by marital status as well as socioeconomic group, we weighted the estimates of smoking prevalence in routine and manual groups by marital status. The survey estimated that while smoking prevalence in the general adult population was 20%, in adults who were single, married or cohabiting the rates were 27%, 14% and 33% respectively (these figures were not available by sex or socio-economic group). We therefore weighted smoking prevalence in men and women in relation to these figures to estimate smoking prevalence in low socioeconomic status adults by sex and marital status (see Table 1).

**Table 1:** **Prevalence of smoking by gender and marital status in routine and manual workers (%)**

	**Base smoking prevalence by gender (before marital status weighting)**	**Smoking prevalence marital status weighting**	**Rate of smoking by sex with marital status weighting**
*Single man*	33	1.35	44.6
*Married man*	33	0.7	23.1
*Cohabitating man*	33	1.65	54.5
*Single woman*	32	1.35	43.2
*Married woman*	32	0.7	22.4
*Cohabitating woman*	32	1.65	52.8

Note: Weightings are the ratio of prevalence by marital status to prevalence in the general population e.g. 27/20 = for single smokers.

### Number of children in poverty by smoking parental marital status and number of children in household

These weighted smoking rates were then applied to estimate the number of children in poverty with smoking parents. For single parent households, we simply applied the smoking rates estimated for single men and women to the number of children in these households. This gave us an estimate of the number of children in poverty living with a smoking single mother or father. For two parent households, we needed to estimate how many contained one smoker and how many contained two. We therefore combined the prevalence data with estimates from an existing study of smoke-free homes and secondhand smoke exposure in children in England by Jarvis et al [20]. This study included a nationally representative sample of 13,365 children, including 695 in 2007 on which our estimates were based. While a more recent estimate based on a larger sample from the whole of the UK would have be preferable, this estimate was the only suitable one available to us, and is likely to be reasonably representative of the whole of the UK. From this we calculated that among parents who smoked in two parent households, 65% were the only smokers, and 35% lived with an adult who also smoked. A worked example of our calculations of the number of children with smoking parents is provided in Additional file 1.

### The cost of smoking in poor households

We estimated the average weekly cost of smoking to poor households by combining data on the number of cigarettes smoked per day by routine and manual workers for men and women with typical costs for manufactured cigarettes and hand rolling tobacco (HRT), both licit and illicit.

Opinions and Lifestyle Survey data indicate that on average, female and male routine and manual workers smoke 12 and 13 cigarettes per day respectively [19]. We estimated the number of packets of 20 cigarettes purchased by low-income manufactured cigarette smokers per week by multiplying the number of cigarettes smoked per day by seven, and dividing by 20; and the number of packets of HRT purchased by low-income HRT smokers per week in the same way, but with the assumption that 50 grams of HRT typically makes approximately 100 cigarettes [21].

To estimate the average weekly spend on manufactured cigarettes and HRT, we combined our estimated weekly quantities purchased with 2012 Tobacco Manufacturers’ Association (TMA) data. This indicates that the average cost of a licit packet of 20 cigarettes was £7.72, and of 50 g HRT £16.11, and that illicit tobacco typically sold for half the price of licit products [22-25].

The proportion of type of cigarettes smoked by sex and age was obtained from the OPN [19] (Opinions and Lifestyle Survey). To make calculations more straightforward, smokers that smoked both packeted cigarettes and HRT were added on to the category they mostly smoked.

In the UK it is estimated that 73% of female and 59% of male smokers smoke mainly manufactured cigarettes (66% of women smoke only packeted, and 6% also smoke HRT, but mainly packeted. 52% of men smoke only packeted, and 7% also smoke HRT but mainly packeted) [19]. HMRC estimates that 7% of packeted cigarettes smoked are illicit, as well as 35% of HRT. Based on these figures, we estimated the proportion of smokers purchasing each type of tobacco (licit packeted, licit HRT, illicit packeted, illicit HRT), and hence the overall average spend on tobacco products.

It should be noted that our estimate is likely to be an overestimate if cheaper licit products, illicit and hand-rolled tobacco are disproportionately consumed by those in poverty.

### Effect on poverty rates of subtracting tobacco expenditure from household income

We estimated the number of children effectively drawn into poverty if parental expenditure on tobacco is subtracted from household income. We calculated how many children are living in a household where the income is above 60% of the median income, but by less than the average spend on tobacco.

The HBAI report provides data on households living between 60% and 70% of the median income; i.e. those living just above the poverty line. We first calculated the number of children who are living in households between 60% and 70% of the median income. We then applied the same method used to calculate the number of children in poverty with smoking parents described above, to estimate the number of children in households between 60% and 70% of the median income with one or two smoking parents.

We calculated the low income thresholds for these income groups for different household structures, which showed that the income difference between these income groups was similar to the average weekly expenditure on tobacco for two smokers calculated in the previous step. We therefore assumed that all children in two-smoker households with a household income between 60% and 70% of the median income would be drawn into effective poverty. Because the spread of the population living between 60% and 70% of the equivalised median is fairly even [1], we also assumed that half of all children between these thresholds with one smoking parent in two-parent households, or one smoking parent in a one-parent household, would be drawn into effective poverty.

## Results

### Number of children living in low income households

Estimated numbers of children in poverty, according to family size and parental marital status, are reported in Table 2, demonstrating that of 2.3 million children living in a poor household in 2011/12, 1.2 million lived with adults who were married or civil-partnered [1].

**Table 2:** **Estimated number of children (thousands) in relative poverty BHC by parental marital status 2011/12 UK**

				**Number of children in relative poverty by number of children in household**			**Total**
				**One**	**Two**	**Three or more**	
**Total number of children by family size**				3,900	6,000	3300	13,200
**% of all children in poverty by family size**				15	15	25	17
**Total number of children in poverty by family size**				585	900	825	2,310
Proportion of all children in poverty in each family size*				0.25	0.39	0.36	1
	All children	% in poverty	Total number of children in poverty				
**Married/Civil Partnered**	8,300	15	1,245	311	486	448	1,245
**Cohabitating**	1,900	20	380	95	148,	137	380
**Single (female)**	2,730	22	601	150	234	216	601
**Single (male)**	270	22	59	15	23,	21	59
**Total**	13,200	17	2,285	571	891	823	2,285

Note: Estimates are rounded to the nearest thousand. A worked example of calculations is provided in the Additional file 1.

*Total number of children in poverty by marital status is multiplied by this proportion to calculate the number of children in poverty by marital status and family size.

### Number of children in poverty in households in which one or more adults smoke

Table 3 shows estimated numbers of children in poor households in which one or two parents smoke, by marital status of the parents and number of children in the household. In total 1.1 million children - almost half of all children in poverty - were estimated to be living in poverty with at least one parent who smokes.

**Table 3:** **Number of children in poverty living with one or more parents who smoke (thousands)**

**Children in poverty living with a single parent who smokes**				
**Number of children in household**	**One Child**	**Two Children**	**Three or more children**	**Total**
**Single mother**	65	101	93	259
**Single father**	6.6	10	9.5	26
**Children living in poverty with two parents of whom at least one smokes**				
**Number of children in household**	**One child**	**Two children**	**Three or more children**	**Total**
**Married Parents, one smokes**	92	143	132	367
**Cohabitating Parents, one smokes**	66	103	95	264
**Married Parents, both smoke**	25	39	36	100
**Cohabitating Parents, both smoke**	18	28	26	72
**Total**	273	424	391	1,088

Note: Estimates rounded to the nearest thousand.

### Expenditure on tobacco

We estimated that a typical woman smoker in relative poverty smokes 84 cigarettes/week, equivalent to 4.2 packs of 20 cigarettes or 0.84 packs of 50 HRT. For men the respective figures were 91 cigarettes, equivalent to 4.55 packs of 20 or 0.91 packs of 50 g HRT. The estimated costs per week of smoking different types of tobacco products to parents in poor households, and the proportion of poor smokers smoking each type of product, are shown in Table 4. Based on our estimates, 68% of female and 55% of male smokers smoke mainly licit packed cigarettes, and spend an average of £32 and £35 on cigarettes per week respectively.

**Table 4:** **Cost of smoking (in pounds) per week for poor smokers**

		**Female**	**Male**
	Average cigarettes smoked per week	84	91
**Licit cigarettes**	Average cost (pack of 20)	7.72	7.72
	Average packs per week	4.2	4.55
	Average weekly spend	32.42	35.13
	Smokers smoking mainly licit packeted cigarettes(%)	68	55
**Licit HRT**	Average cost (50 g)	16.11	16.11
	Average packs per week	0.84	0.91
	Average weekly spend	13.53	14.66
	Smokers smoking mainly licit HRT (%)	18	27
**Illicit cigarettes**	Average cost (pack of 20)	3.86	3.86
	Average packs per week	4.2	4.55
	Average weekly spend	16.21	17.56
	Smokers smoking mainly illicit packeted cigarettes (%)	5	4
**Illicit HRT**	Average cost (50 g)	8.05	8.05
	Average packs per week	0.84	0.91
	Average weekly spend (£)	6.72	7.33
	Smokers smoking mainly illicit HRT (%)	9	14
**Overall average spend on tobacco (£)**		25.90	25.01

### Number of children ‘drawn into poverty’ by parental smoking expenditure

According to our estimates, there are nearly 4 million children living in households below 70% of the median income, and 1.6 million children live in households where the income is between 60% and 70% of the median income (Table 5).

**Table 5:** **Estimated number of children below 70% and between 60% and 70% of median income (thousands)**

**Marital status**	**All children**	**% children below 70% median income**	**Children below 70% median income by number of children in household**			**Total children below 70% median income**	**Children between 60% and 70% median income by number of children in household**			**Total children between 60% and 70% median income**
			**One**	**Two**	**Three or more**		**One**	**Two**	**Three or more**	
**Married/Civil Partnered**	8,300	24	458	797	737	1,992	147	311	289	747
**Cohabitating**	1,900	33	144	251	232	627	49	103	95	247
**Single (female)**	2,730	42	264	458	424	1,146	113	224	208	545
**Single (male)**	270	42	26	45	42	113	11	22	21	54
**Total**	13,200	29	892	1,551	1,435	3,878	321	660	612	1,593

Note: Estimates rounded to the nearest thousand.

Estimates reported in Table 6 suggest that three quarters of a million children living in households with an income between 60% and 70% of the median income are living with at least one smoker. Given the differences in income between the 60% and 70% thresholds (shown in Additional file 1), we have estimated that over 432,000 children may be viewed as having been drawn into poverty by parental smoking.

**Table 6:** **Number of children likely to be drawn into poverty by parental smoking (thousands)**

**Children with a single parent who smokes**					
**Number of children in household**	**One Child**	**Two Children**	**Three or more children**	**Total**	**Total drawn into poverty**
**Single mother**	49	97	90	236	118
**Single father**	5	10	9	24	12
**Children with two parents of whom at least one smokes**					
**Married, one smokes**	43	92	85	220	110
**Cohabitating, one smokes**	34	71	66	172	86
**Married, both smoke**	12	25	23	60	60
**Cohabitating, both smoke**	9	19	18	47	47
**Total**	99	207	192	758	432

Assumptions: If all parents in household smoke their children are drawn into poverty by expenditure on cigarettes. Half of children living with two parents, one of whom smokes, are drawn into poverty.

Note: Estimates rounded to the nearest thousand.

## Discussion

Our study suggests that approximately 1.1 million children, or nearly half of all children in relative poverty in 2012, had at least one smoking parent. We also estimate that around 432,000 children would be classed as being in poverty if parental tobacco expenditure were subtracted from household income. Thus there may be over 1.5 million children living in circumstances of severe financial deprivation whose plight is exacerbated by parental smoking. Our study thus identifies a key opportunity and priority for government action to reduce the number of children experiencing the adverse effects of poverty through measures that encourage parents and carers, particularly those in low income groups, to quit smoking.

The failure to meet the government target on child poverty means that measures to alleviate the effects of poverty are more important than ever. In this study we have addressed a contributor to child poverty that has not, to our knowledge, previously been quantified in this context and falls outside standard child poverty statistics. Effective tobacco control interventions which enable low income smokers to quit, can thus potentially play an important role in reducing the burden of child poverty, and may improve child health and wellbeing by more than just the removal of direct effects of tobacco smoke. Recent reviews suggest that price increases are the intervention with the greatest potential for reducing socioeconomic disparities in smoking [26,27]. However, price rises must be coupled with accessible individual-level smoking cessation support – which can be funded, at least to some extent, from tobacco tax revenues - to help counter the effect of price increases on low-income smokers who continue to smoke: they will spend a larger proportion of their income on smoking than higher-income groups [28].

Our estimates are inevitably approximate as constraints in data availability have required us to make a number of assumptions; however we ensured that such assumptions were conservative so our findings are likely to underestimate the true figures. In addition, our analyses are subject to aggregation error. The estimates of smoking prevalence applied in this study were based on self-report, and may therefore underestimate true prevalence [19]. Since smoking rates for adults in poverty are not available from national survey reports, including the census, we have made the assumption that smoking rates in this group will be at least as high as those in routine and manual workers, a group for which suitable data are available. It is likely however that smoking rates are higher in the most deprived [19], though there is evidence of a good correlation between these two groups [29], with the consequence that this assumption is likely to underestimate true smoking prevalence in poor adults and hence the proportion of children in poverty with smoking parents. Estimates of smoking prevalence were not available by socio-economic group and marital status, so we have had to weight smoking prevalence in the routine and manual group using estimates of smoking prevalence by marital status from the general population. Our estimate of the cost of smoking licit tobacco is based on the recommended retail price (RRP) of a typical pack of 20 cigarettes in the Most Popular Price Category (MPPC), but in practice it is likely that many poor smokers smoke lower-cost manufactured cigarettes, resulting in some overestimation of cost in our study. However we have also assumed that the proportion smoking illicit tobacco, priced at half that of licit product, is the same in low income groups as in the general population, which is almost certainly an underestimate. Detailed data on the income distribution in households were not available to us, and we have therefore used the number of children between 60% and 70% of the median income, and the differences between these income thresholds, to estimate the number of children drawn into poverty by parental tobacco expenditure.

Our estimates suggest that low-income smokers who smoke an average of 12–13 cigarettes per day (the national average in routine and manual workers) will spend over £13 per week (£700 per year) if they smoke licit HRT, and around £32-£35 (£1600-1800 per year) if they smoke manufactured cigarettes, although it seems likely that people in poverty buy more HRT and illicit tobacco than other smokers. When we consider that the poverty threshold level of income (60% of median income BHC) for a single parent household with one child under 14 is £223, and for a two parent household with two children is £392, it is clear that this spend represents a substantial proportion of income in these households - at least 4% for a cigarette smoker in a two-parent, two-child household even if the smoker smokes illicit tobacco - especially if both parents are smokers [30]. Furthermore, many households below the poverty line will be earning incomes well below these thresholds, with poverty exacerbated by expenditure on smoking.

Despite inaccuracies in our estimates, however, our findings indicate that implementing measures that reduce the prevalence of smoking among low socioeconomic status groups would not only improve health but also relieve poverty. Use of tax to reduce the affordability of tobacco products, particularly of lower cost cigarettes and hand-rolling tobacco, along with measure to reduce availability of illicit supplies are key if counterintuitive policies, since low socioeconomic groups are highly responsive to price increases [31,32].

## Conclusions

Given public sensitivity over the use of welfare benefits by the poor and long-standing caricatures of the deserving and undeserving poor, care is required to avoid moralising and imposing population-level utility values on a group living with very different stressors and challenges to the majority of the population. Nonetheless, it is clear from our estimates that smoking places a significant additional financial burden on large numbers of children living in low-income households, and that governments have a duty to ensure that tobacco control policies are fully implemented to minimise this effect. Both the ethical and practical challenges associated with conducting this type of study serve to underline the importance of further detailed research. The use (and in some cases collection) of more detailed data to maximise the accuracy of estimates, as well as the consideration of other types of poverty such as persistent poverty, subjective poverty and material deprivation will enable us to more fully understand the substantial burden of smoking on poor households.

## Additional file

Additional file 1:
**Table showing mean low-income thresholds for different household compositions.** Worked examples of calculations.
