# Supplementary material for: Parental smoking and child poverty in the UK: an analysis of national survey data
Source: BMC Public Health. 2015 May 29;15:507. doi: 10.1186/s12889-015-1797-z (PMC4448212; doi:10.1186/s12889-015-1797-z)
Supplement: Additional file 1: — Table showing mean low-income thresholds for different household compositions. Worked examples of calculations. [file 12889_2015_1797_MOESM1_ESM.docx]

**Parental smoking and child poverty: Appendices**

***Appendix 1: Calculating the number of children in poverty by parental marital status and family size (Based on data from HBAI 2011/12)***

*Example*

*Step 1*

1. To calculate the number of children in poverty living in a household with a married/civil partnered couple
2. Total number of children living with a married/civil partnered couple = 8,300,000
3. Percentage of children in married/civil partnered households below 60% of median income = 15%
4. Number of children in poverty living in a household with a married/civil partnered couple = 8,300,000 x 0.15 = 1,245,000

*Step 2*

1. To calculate the number of children in poverty living in a household with a married/civil partnered couple and three or more children
2. Number of children in poverty living in a household with a married/civil partnered couple = 1,245,000
3. Total number of children in households with three of more children = 3,300,000
4. Percentage of children in households with three or more children below 60% of median income =25%
5. Total number of children with three or more children below 60% of median income = 825,000
6. Total number of children in poverty = 2,310,000
7. Proportion of children in poverty in households with three or more children = 825,000/2,310,000 = 0.36
8. Number of children in poverty living in a household with a married/civil partnered couple and three of more children = 1,245,000 x 0.36 = 448,200

***Appendix 2: Calculating the number of children in poverty by smoking parental marital status and number of children in household***

*Examples*

*Step 1 – Single parent households*

1. Number of children in poverty living in households with a single female parent and no siblings = 150,150
2. Smoking prevalence in poor single females = 43.2%
3. Number of children in poverty in households with a single female parent who smokes and no siblings = 150,150 x 0.432 = 64,865

*Step 2 – Two parent households*

1. Number of children in poverty living in households with married parents and no siblings = 311,250
2. Smoking prevalence in poor married women =22.4%
3. Smoking prevalence in poor married men = 23.1%
4. Number of children in poverty living in households with married parents and no siblings, with a smoking mother = 311,250 x 0.224 = 69,720
5. Number of children in poverty living in households with married parents and no siblings, with a smoking father = 311,250 x 0.231 = 71,899
6. Total number of children in two-parent household = 141,619
7. Based on Jarvis et al, among parents who smoke in two parent households, 65% are the only smokers, and 35% live with an adult who also smokes.
8. Total number of children in two parent households with 1 smoking parent = 141,619 x 0.65 = 91,783 (Note: estimates are subject to rounding error)
9. Total number of children in a two parent households with 2 smoking parents = (141,619 x 0.35) / 2 (divided by two to avoid double counting, because the unit we are interested in is children)

**Appendix 3: Mean low-income thresholds for different household compositions (£)**

| **Household composition** | **Median** | **60%** | **70%** | **Difference between 60% and 70% thresholds** |
| --- | --- | --- | --- | --- |
| *Single parent, 1 child u14* | 372 | 223 | 260 | 37 |
| *Couple, 1 child u14* | 513 | 308 | 359 | 51 |
| *Single parent, 1 child 14+* | 427 | 256 | 298 | 42 |
| *Couple, 1 child 14+* | 568 | 341 | 398 | 57 |
| *Single parent, 2 children (1 u14, 1 14+)* | 513 | 308 | 359 | 51 |
| *Couple, 2 children (1 u14, 1 14+)* | 653 | 392 | 457 | 65 |
| *Single parent, 3 children (2 u14, 1 14+)* | 598 | 359 | 419 | 60 |
| *Couple, 3 children (2 u14, 1 14+)* | 740 | 444 | 518 | 74 |
| *Single parent, 4 children (2 u14, 2 14+)* | 740 | 444 | 518 | 74 |
| *Couple, 4 children (2 u14, 2 14+)* | 880 | 528 | 616 | 88 |
